# Adverse childhood experiences and mental health in young adults: a longitudinal survey

**DOI:** 10.1186/1471-2458-7-30

**Published:** 2007-03-07

**Authors:** Elizabeth A Schilling, Robert H Aseltine, Susan Gore

**Affiliations:** 1 Institute for Public Health Research, University of Connecticut, 99 Ash Street, East Hartford, Connecticut, 06108 USA; 2 Division of Behavioral Sciences and Community Health, University of Connecticut Health Center, 263 Farmington Road, Farmington, Connecticut 06030-3910, USA; 3 Department of Sociology, University of Massachusetts–Boston, 100 Morrissey Boulevard, Boston, Massachusetts 02125, USA

## Abstract

**Background:**

Adverse childhood experiences (ACEs) have been consistently linked to psychiatric difficulties in children and adults. However, the long-term effects of ACEs on mental health during the early adult years have been understudied. In addition, many studies are methodologically limited by use of non-representative samples, and few studies have investigated gender and racial differences. The current study relates self-reported lifetime exposure to a range of ACEs in a community sample of high school seniors to three mental health outcomes–depressive symptoms, drug abuse, and antisocial behavior–two years later during the transition to adulthood.

**Methods:**

The study has a two-wave, prospective design. A systematic probability sample of high school seniors (N = 1093) was taken from communities of diverse socioeconomic status. They were interviewed in person in 1998 and over the telephone two years later. Gender and racial differences in ACE prevalence were tested with chi-square tests. Each mental health outcome was regressed on one ACE, controlling for gender, race/ethnicity, and SES to obtain partially standardized regression coefficients.

**Results:**

Most ACEs were strongly associated with all three outcomes. The cumulative effect of ACEs was significant and of similar magnitude for all three outcomes. Except for sex abuse/assault, significant gender differences in the effects of single ACEs on depression and drug use were not observed. However, boys who experienced ACEs were more likely to engage in antisocial behavior early in young adulthood than girls who experienced similar ACEs. Where racial/ethnic differences existed, the adverse mental health impact of ACEs on Whites was consistently greater than on Blacks and Hispanics.

**Conclusion:**

Our sample of young adults from urban, socio-economically disadvantaged communities reported high rates of adverse childhood experiences. The public health impact of childhood adversity is evident in the very strong association between childhood adversity and depressive symptoms, antisocial behavior, and drug use during the early transition to adulthood. These findings, coupled with evidence that the impact of major childhood adversities persists well into adulthood, indicate the critical need for prevention and intervention strategies targeting early adverse experiences and their mental health consequences.

## Background

Adverse childhood experiences (ACEs) have been consistently linked to psychiatric difficulties in children and adults [1-20]. Much of the research documenting these associations has been performed on clinical and/or cross-sectional samples; only recently have these associations been documented in large, community samples [2,6,12-18,20]. A notable recent series of research studies on this topic has linked ACEs to a range of mental health outcomes well into adulthood (e.g., [12,14,17,18]) However, very few studies have investigated the association of ACEs with mental health in early adulthood. The current study investigates the prevalence of a variety of lifetime ACEs reported by a sample of racially and economically diverse high school seniors, and estimates the impact of these experiences on three mental health outcomes–depression, drug use, and antisocial behavior–assessed two years later. In addition, this study investigates under-explored gender and racial/ethnic differences in these associations.

Despite the substantial body of research tying childhood experiences to adult mental health, the paucity of studies examining the effects of ACEs during the years following high school constitutes a weak link in the developmental and epidemiological literature. For many youths, this is a tumultuous time, as evidenced by a higher frequency of exposure to major life events [21,22] and higher rates of mental disorder than at any other life stage [23,24]. Mental health consequences of ACEs may disrupt these normal developmental processes [19], increasing the risk of poor adult adjustment.

The possibility that gender and race/ethnicity may moderate the impact of ACEs on mental health during young adulthood has not received much attention in the research literature. This may be because low prevalences for many childhood ACEs make subgroup analyses difficult. We are aware of only a handful of studies that have investigated gender differences in young adult samples [2,8,9]. Compared to gender, even less is known about racial/ethnic differences in the mental health impact of ACEs, despite the possibility that cultural context may modify their effects [25-27]. Most previous samples have included limited ethnic diversity (e.g., [8,20]), or have been too small to investigate such effects.

An additional limitation of existent research on the mental health impact of ACEs involves the limited number of outcomes examined. As Kessler et al. [2] have noted, few studies have examined multiple mental health outcomes simultaneously and most literature on the impact of victimization (other than child abuse) is limited to PTSD (see [19]). The possibility of specificity in the effects of ACEs on mental health renders investigations limited to a single mental health dimension problematic because the absence of an adversity-outcome association for one type of outcome may falsely imply that no associations exist for other outcomes [28,29]. Similarly, although a few studies have included a range of events experienced in childhood and adolescence [2,8,9,12-18,20], the number of ACEs assessed in most studies is limited. Thus, comparisons of the effects of various events across mental health outcomes are difficult to make. To our knowledge, only six studies [2,6,10,11,13,16], have investigated the effects of a variety of ACEs on a variety of psychiatric outcomes; in general, little specificity in ACE-outcome associations was found.

Our study aims to advance our understanding of the effects of lifetime trauma and adversity on the mental health of young adults transitioning from high school into the wider world. In addition to the advantage of focusing on a specific developmental period, this study has methodological advantages over many previous studies. First, because our sample consists of high school seniors systematically selected from a set of racially and economically diverse communities, it contains a natural cohort-matched comparison group. Most previous studies of ACEs and mental health are compromised by the lack of such a comparable comparison group [7]. Second, the diverse nature of the sample provides a frequency of rare ACEs high enough to reliably assess the effects of individual ACEs on mental health outcomes, and to investigate gender and racial/ethnic differences. Third, our sample includes a large number of participants of one age group with the result that age is not confounded with base rates of psychiatric disorder, lifetime exposure to trauma, or other social variables such as employment or marital status [23,29]. Finally, the late adolescent period may be the optimal time to obtain a comprehensive self-report of lifetime trauma and adversity in the pre-adult years in terms of accuracy of memory, as Perkonigg et al. have discussed [30]. Thus, our study addresses a gap in current knowledge by combining methodological advantages of studying the association of ACEs with mental health in early adulthood with the importance of this developmental period.

## Methods

### Sample and procedures

The data for these analyses come from the first two waves of a prospective study of childhood experiences, adolescent development, and mental health among students in 7 communities/school districts in the Boston Massachusetts metropolitan area. The communities represented were selected from the Boston CMSA (Consolidated Metropolitan Statistical Area), which defines a large, contiguous geographic area in eastern Massachusetts and includes major cities whose schools were selected to insure the sample would reflect diversity in family socioeconomic background and ethnicity, as well as diversity among the students in post-high school educational and work pathways. This study was approved by the University of Massachusetts-Boston Internal Review Board.

A systematic probability sample of 1,578 high school seniors from 9 public schools serving these communities was selected using official rosters obtained from each school. Students were sampled proportionate to the size of the high school they were attending. A total of 1143 of these students were interviewed in the winter and spring of 1998, representing a 72% response rate. Interviews were also conducted with former students of these schools who would have been in the senior cohort but who dropped out. School systems provided initial lists of former students who met the criteria for dropping out: they had left school prior to graduation and had not transferred to another high school within 6 months, or had taken a temporary leave of absence. Efforts to contact these former students yielded the estimate that only 2/3 of the individuals on our lists actually met the dropout criteria described above. Interviews were completed with 182 students, resulting in an estimated response rate of 70%.

At Time 1, personal interviews, averaging 70 minutes in length, were conducted by trained professional interviewers from the University of Massachusetts Center for Survey Research. A total of 66 interviews were done over the phone for individuals who were not available for a personal interview. Passive parental consent was obtained from parents following a home mailing of study information including a letter from the school indicating their support. Students gave their consent for participation at the time of the interview and were given a gift of two movie tickets for their time.

The second wave of interviews was conducted in 2000 and involved 1093 members (83%) of the Time 1 sample, which includes both the graduates and dropouts. This interview was conducted over the telephone with all individuals, with verbal consent given at that time. Participants were given a check for $50 in appreciation for their time. Attrition from the sample was largely a result of an inability to trace respondents due to relocation. Only 3% (n = 52) of the Time 1 sample refused to participate in the follow-up; in addition, less than 1% (n = 12) of participants did not participate in the follow-up because of the refusal of a parent or other proxy. We examined variables associated with study retention through estimating a logistic regression model that included dummy variables for race/ethnicity (with whites the omitted category), gender, dropout status, parents' highest education, family standard of living, depressed mood, and family support. Significant predictors of study attrition (all p < .001) included: being Black or Hispanic, dropout status, and having less educated parents (p < .001). Specifically, there was a 12% attrition rate among Whites, in contrast with a 31% rate among Hispanics, and from 18 to 22% among the other race/ethnic groups. The attrition rate for the dropout subsample was estimated on the basis of a projected eligibility rate of 2/3, based on the numbers of individuals who were designated as dropouts in our screenings. Therefore we report an estimated attrition rate of 30%, which includes 14% refusals and 16% non-interviews due to an inability to make contact after an average of 23 tries.

A demographic profile of the sample is presented in Table 1. As a whole the sample is quite diverse and contains large numbers of youths from disadvantaged backgrounds. Half of the sample is non-White, and 44% of participants have parents with a high school education or less. A quarter of respondents have participated in an English as a Second Language (ESL) or bilingual educational program at any time in their schooling, and roughly a third report that they qualify for free or reduced-price school lunches. Although not shown in Table 1, significant differences in SES are apparent among the three racial/ethnic categories examined in this study [31].

**Table 1:** Demographic information at Wave 1

	Total			Total	
	n	%		n	%

Gender			Parents' Education		
Male	643	48%	Less than High School	178	14%
Female	682	52%	High School Graduate^a^	483	38%
Age			Some College	218	17%
16–17	396	35%	College Degree or Above	401	31%
18	559	49%	ESL		
19	149	13%	ESL	313	24%
20 and above	39	2%	No ESL	1011	76%
Family Structure			Race/Ethnicity		
Intact Two Parent	666	50%	White	648	49%
Step-Parent	139	11%	Black	279	21%
Single Parent	390	29%	Hispanic	145	11%
Other	130	10%	Asian	93	7%
			Multi	71	5%
			Other	89	7%

^a ^or Voc/Other non-college

### Measures

Three dependent mental health variables were used in this analysis: depressed mood, frequency of drug use, and frequency of antisocial behavior. Each outcome variable was standardized to have a mean of 0 and a standard deviation of 1. Depressed mood was measured with a modified 12-item version of the 20- item Center for Epidemiological Studies' Depression (CESD) scale [32]. The measure has an internal consistency of .81 at Time 1 and .82 at Time 2. Antisocial behavior was assessed by asking respondents to report the number of times in the past 12 months that they had participated in 14 types of aggressive and/or illegal behavior. Prior to calculating a summary measure, frequencies of each item were truncated to 10, in order to diminish the effect of outliers. Drug use was assessed with a self-administered form assessing frequency of (a) illegal drugs used or (b) legal drugs used without a doctor's prescription, in larger amounts than prescribed, or for a longer period of time than prescribed. Respondents were asked how many times in the past 12 months they had used each drug, from "never" to "more than 10 times," and a summary variable was created indicating the mean frequency of use. Missing data for the summary measures were excluded from analyses, resulting in an effective sample size ranging between 1018 and 1091 for these analyses.

Respondent's self-identified race and ethnic background was obtained in the Time 1 interview by using the two-pronged approach used in the 2000 Census. We first asked respondents whether they considered themselves to be of Hispanic or Latino origin, followed by a question about race which allowed for the selection of one or more options. In coding a single measure of race/ethnic identity, we maintained as a group all individuals who said they were of Hispanic or Latino background, irrespective of their racial designation. From these variables, we were able to identify 5 race/ethnic groups for analyses: Whites, Hispanics, Blacks, Asian Americans, and multi-ethnic youth.

Participants answered a series of questions regarding ACEs that ever happened in his/her life. The content and structure of these items are identical to items used previously by [13] and in the NCS (e.g., [2]). The items included (a) Did your parents ever have a marital separation of one month or more? (b) Were you ever sent away from home because you did something wrong or your parents felt they couldn't handle you? (c) Did your father or mother not have a job for a long time when they wanted to work? (d) Did either of your parents drink or use drugs so often or so regularly that it caused problems for the family? For items (e) through (l) participants were asked "did this ever happen to you?" (e) You witnessed someone being badly injured or killed? (f) You were seriously physically attacked or assaulted? (g) You were physically abused as a child? (h) You were seriously neglected as a child? (i) You were threatened with a weapon, held captive, or kidnapped? (j) You witnessed someone being badly injured or killed? For each of the above questions, dummy variables were created, coded as "1" if the participant answered "yes" and "0" if the participant answered "no."

In addition, to assess sexual abuse/assault events, participants were asked if they had ever been: (j) raped (someone had sexual intercourse with you when you did not want to by threatening you or using some degree of force) (k) sexually molested (someone touched or felt your genitals when you did not want them to) and/or (l) sexually abused as a child. To prevent duplication in the reporting of sexual victimization, respondents were instructed to exclude previously reported events in answering the latter two questions (k and l). The effects of these events on the three mental health outcomes were assessed for each event individually in preliminary analyses. Because of the overlap among these items due to the way these questions were structured, and due also to the similarity in their effects on each outcome, a participant who answered "yes" to any of these outcomes was coded "1" on a single dummy variable. A participant was coded "0" on the sexual abuse/assault variable if they answered no to all three questions.

Finally, recent research indicates that multiple ACEs increase the risk of experiencing psychiatric disorder [11-15,33]. To make a preliminary estimate of the cumulative effect of ACEs on mental health in young adulthood, we employed the sum of the dummy variables described above. To limit skew, this variable was truncated at 5.

### Overview of analysis

In the first set of analyses, ACE prevalences were calculated for the entire sample and within gender and race/ethnic categories. Chi-square tests were performed to determine whether ACE prevalences differed by race/ethnicity or gender.

The second set of analyses involved estimating the effects of ACEs reported at Wave 1 on the three mental health outcomes assessed at Wave 2. Wave 2 mental health outcomes were used in an effort to minimize recall bias related to the selective memory of persons in depressed affective states for lifetime stressful events [34]. Three sets of regressions were performed. The first set included separate regressions of each mental health outcome on each ACE. The second set included a test of gender differences in effects of each ACE on the outcome measures. The final set included a test of differences in the effects of ACEs on mental health for Whites, Blacks, and Hispanics for participants in those racial/ethnic groups. All regressions included a control for parents' education as an indicator of SES.

Multiple comparisons were handled by ensuring that the number of significant tests in a set of analyses exceeded those expected by chance at an alpha level of .05 before interpreting individual effects. For example, we conducted 30 regressions to investigate race/ethnicity effects (10 ACEs with 3 outcomes). At the .05 alpha level, we would expect that 1 to 2 sets of ACE X race/ethnicity interactions would be significant. In each case, the number of significant differences exceeded those expected by chance by a wide margin.

## Results

### ACE prevalences

Table 2 includes prevalence information for lifetime experience of the ten childhood adversities, broken down by gender and race/ethnicity. Only the three racial/ethnic groups with substantial subsample sizes (Whites, Blacks and Hispanics) are reported. The most commonly reported ACE was parental separation (27.5%). At least 1 out of 7 respondents reported experiencing (a) an unemployed parent, (b) a parent with a drinking or drug problem, or (c) witnessing injury or murder. Girls reported much higher rates of sexual abuse/assault events than boys (11.7% vs. 1.4%). Girls also reported higher rates of physical abuse and serious neglect. However, boys reported higher rates of (a) witnessing an injury/murder (19.7% v. 13.8%), (b) physical assault (10.8% v. 4.5%), and (c) being threatened, held captive, or kidnapped (15.5% v. 4.3%).

**Table 2:** Prevalence of adverse childhood experiences (ACEs) reported by high school seniors

ACE	Girls	Boys	White	Black	Hispanic	Total
	%	%	%	%	%	%

Parents separated	27.9	27.0	28.0	29.0	29.3	27.5
Sent away from home	5.2	5.0	5.8	4.1	6.0	5.1
Parent unemployed	18.3	16.9	17.5	22.4	18.2	17.6
Parent drink/drug problem ^Race^	15.8	12.4	16.8	11.4	13.1	14.2
Witnessed injury/murder ^Sex, Race^	13.8	19.7	12.9	24.3	25.0	16.6
Sex abuse/assault ^Sex, Race^	11.7	1.4	5.9	5.9	13.1	6.8
Physically assaulted ^Sex^	4.5	10.8	8.6	5.9	8.0	7.5
Physically abused	5.0	3.3	4.5	4.1	6.0	4.2
Seriously neglected ^Sex^	3.3	1.7	2.3	3.7	2.0	2.6
Threatened/captive ^Sex, Race^	4.3	15.5	11.2	5.9	16.0	9.6
Total number	575	518	573	219	100	1093

Sex and race superscripts indicate prevalences differ by these background characteristics at the .05 alpha level.

Significant racial/ethnic differences are also evident in the prevalence data in Table 2. Approximately 1 in 6 White respondents reported that one of their parents had a drinking or drug problem, a rate approximately 50% higher than the rate reported by Blacks, and 30% higher than the rate reported by Hispanics. 1 in 4 Black and Hispanic respondents reported witnessing a serious injury or murder, a rate twice as high as that reported by Whites. Finally, about 1 in 6 Hispanics reported being threatened with a weapon, held captive, or kidnapped. This rate was 43% higher than the rate reported by Whites and almost two times higher than the rate reported by Blacks.

### Effects of ACEs on mental health

The effects of lifetime ACEs reported at Wave 1 on mental health outcomes at Wave 2 are presented in Table 3. This table presents partially standardized coefficients from regression models in which each outcome was regressed on one ACE, controlling for gender, race/ethnicity, and SES. As a result, the coefficients for each individual ACE can be interpreted directly as effect sizes reflecting the magnitude of these associations [35]. All ACEs had some impact on at least one mental health outcome, and the impact tended to vary more across ACEs for the same outcome than across outcomes for the same ACE. Eight of the ten ACEs were significantly related to reports of higher depressive symptoms. Only the two most frequently experienced ACEs–parents separating and a parent unable to find work–were not significantly related to depressive symptoms. Nine of ten ACEs were significantly related to drug use, and eight of ten ACEs were significantly associated with antisocial behavior. All effect sizes reported in the text below are significant at an alpha level of .05.

**Table 3:** Individual and cumulative effects of ACEs on standardized mental health outcomes

ACE	CESD		Drug Use		Antisocial Behavior	
	b.	s.e.	b.	s.e.	b.	s.e.

Parents separated	.126	.067	.135*	.068	.071	.067
Sent away from home	.602*	.135	.808*	.138	.984*	.133
Parent unemployed	.134	.079	.180*	.080	.164*	.079
Parent drink/drug problem	.402*	.086	.332*	.088	.377*	.086
Witnessed injury/murder	.275*	.081	.329*	.083	.542*	.080
Sex abuse/assault	.698*	.120	.441*	.123	.291*	.122
Physically assaulted	.562*	.114	.627*	.117	.732*	.113
Physically abused	.450*	.149	.278	.153	.443*	.150
Seriously neglected	.520*	.189	.401*	.189	.306	.190
Threatened/captive	.435*	.103	.524*	.107	.333*	.104
Any ACE	.260*	.061	.267*	.062	.219*	.062
Sum of ACEs	.158*	.021	.165*	.022	.168*	.021

*p < .05

Each model includes as predictors only 1 ACE and includes controls for the effects of gender, race, and parents' SES.

Effects of ACEs across mental health outcomes were generally similar for a given ACE. As is evident from the coefficients in Table 3, the effect of experiencing at least one ACE ("Any ACE") and the ACE cumulative effect were significant and of similar magnitude for all 3 outcomes. Increasing ACE frequency was significantly associated with increasing impact on depressive symptoms (b = .158), drug use (b = .165), and antisocial behavior (b = .168). This effect becomes quite strong when a respondent has experienced multiple ACEs. For example, respondents who experienced four ACEs scored, on average, approximately 1 standard deviation higher on each outcome than persons who had experienced no ACEs.

### Gender differences

As a prelude to examining gender differences in the effects of ACEs on mental health, we note that girls and boys differed significantly in average levels of drug use and antisocial behavior. Boys, compared to girls, reported more frequent drug use (M = 1.28, SD = .51 vs. M = 1.20, SD = .43; data not shown) and antisocial behavior (M = .22, SD = .62 vs. M = .06, SD = .33). The mean level of depressive symptoms did not differ by gender, however (M = 1.69, SD = .48 vs. 1.74, SD = .52, respectively).

Table 4 presents gender differences in the effects of ACEs at Wave 1 on mental health outcomes at Wave 2. Sex abuse/assault was the only ACE in which effects differed by gender for all three outcomes. In each case, sexual victimization was associated with more depressive symptoms, drug use, and antisocial behavior in boys than girls. Even though few boys experienced sexual victimization, it appears to have an especially large impact on boys' mental health (b = 1.41 for depressive symptoms, b = 1.85 for drug use, and b = 1.06 for antisocial behavior). Except for sex abuse/assault, significant gender differences in the effects of single ACEs on depression and drug use were not observed. However, the cumulative effect of ACEs on drug use was significantly greater for boys (b = .206) than girls (b = .130). Boys with 5 ACEs scored 1 standard deviation higher on drug use than boys experiencing no events. For girls, experiencing more than 7 ACEs resulted in the same increase in drug use. The impact of six ACEs–being sent away from home, witnessing an injury or murder, sexual abuse/assault, physical assault, physical abuse, and serious neglect–had stronger effects on antisocial behavior in boys than on girls. In fact, three severe ACEs–sexual abuse/assault, physical abuse, and serious neglect–were not associated with antisocial behavior in girls. The cumulative effect of ACEs on antisocial behavior was also significantly greater for boys (b = .248) than girls (b = .094). Boys with 4 ACEs scored 1 standard deviation higher on antisocial behavior than boys experiencing no ACEs. For girls, experiencing more than 10 ACEs resulted in the same increase in antisocial behavior. These data indicate that boys who experience ACEs are much more likely to engage in antisocial behavior than girls who experience similar ACEs.

**Table 4:** Effects of ACEs separately by gender on standardized mental health outcomes with significant gender effects

ACE	Girls		Boys	
	b.	s.e.	b.	s.e.

Depression				
Sex abuse/assault	.614*	.127	1.41*	.370
Drug Use				
Sex abuse/assault	.285*	.128	1.85*	.388
Sum of ACEs	.130*	.029	.206*	.032
Antisocial Behavior				
Sent away from home	.522*	.180	1.52*	.194
Witnessed injury/murder	.322*	.118	.723*	.107
Sex abuse/assault	.199	.129	1.06*	.376
Physically assaulted	.586*	.196	.803*	.137
Physically abused	.070	.188	1.06*	.242
Seriously neglected	.017	.230	.937*	.333
Sum of ACEs	.094*	.029	.248*	.030

*p < .05

Only significant gender differences are presented. All models include controls for main effects of gender, race, and parents' SES.

### Race and ethnic differences

Regarding race and ethnic differences, again we begin by noting that mean levels of drug use and depressive symptoms differed among Whites, Blacks, and Hispanics. Hispanics reported the highest depressive symptoms (M = 1.88, SD = .55) followed by Blacks (M = 1.80, SD = .52) and Whites (M = 1.65, SD = .49). Whites reported the highest levels of drug use (M = 1.34, SD = .57) followed by Hispanics (M = 1.13, SD = .31) and Blacks (M = 1.08, SD = .15). The frequency of antisocial behaviors did not differ between these three groups: Whites (M = .14, SD = .53), Blacks (M = .14, SD = .52), Hispanics (M = .12, SD = .31).

Table 5 presents racial/ethnic differences in the effects of ACEs among Whites, Blacks, and Hispanics. These data indicate that the mental health effects of a number of ACEs are consistently stronger among Whites than among Blacks or Hispanics. In fact, the cumulative effect of adversity as well as three individual ACEs–(a) sent away from home, (b) parent with drinking/drug problem and, (c) being threatened with a weapon or held captive–are significantly associated with drug use among Whites only. Whites with 4 ACEs scored almost 1 standard deviation higher on drug use than Whites experiencing no ACEs. Blacks and Hispanics would need to experience more than 20 ACEs to report the same increase in drug use. Similarly, having a parent who (a) was unable to find work for a long time or who (b) had a drinking/drug problem was significantly associated with antisocial behavior in Whites only. Witnessing a severe injury or murder was most strongly associated with both depression and antisocial behavior in Whites. It was not significantly associated with either outcome in Hispanics, and with only antisocial behavior in Blacks.

**Table 5:** Effects of ACEs by race on standardized mental health outcomes with significant race differences

ACE	White		Black		Hispanic	
	b.	s.e.	b.	s.e.	b.	s.e.

Depression						
Witness injury/murder	.565*	.124	.004	.156	.289	.230
Drug Use						
Sent away from home	1.06*	.187	.163	.362	.027	.424
Parent drink/drug problem	.480*	.116	-.066	.220	-.098	.340
Threatened/captive	.705*	.141	.066	.314	-.122	.313
Sum of ACEs	.224*	.029	.029	.054	.048	.083
Antisocial Behavior						
Parent unemployed	.318*	.110	-.212	.163	.096	.262
Parent drink/drug problem	.492*	.112	-.006	.212	-.199	.296
Witnessed injury/murder	.743*	.123	.414*	.155	.053	.228

*p < .05

Only significant race/ethnic differences are presented. All models include controls for main effects of race/ethnicity, gender, and parents' SES.

## Discussion

Our results clearly demonstrate that a large percentage of young people from at-risk communities enter adulthood with serious adversity in their pasts. High school seniors in this sample suffered high levels of exposure to ACEs during their lifetimes, most likely due to the urban, socio-economically disadvantaged character of the communities in this study. Rates of most ACEs reported by girls and boys in this sample exceed estimates of ACEs occurring before age 16 in the NCS sample [2], in a rural North Carolina (NC) sample [20], and in a mostly white, lower middle class sample [8], although they appear to be lower than those in the Singer et al. [6] high school sample. In addition, our results highlight the importance of race as a risk factor for exposure to certain types of ACEs. The higher prevalence estimates reported in the present study are almost certainly a function of the greater racial/ethnic diversity relative to previous studies (e.g. [22,36]).

The public health impact of such high levels of exposure to childhood adversities is evident in their strong and pervasive affects on mental health in early adulthood. Substantial effect sizes for events such as sexual abuse/assault and physical abuse and assault were observed, providing further evidence of the especially pernicious effects of child maltreatment and violence on mental health [3,6,11]. In addition, the pervasive nature of these effects–only 1 of the 10 ACEs examined was not significantly associated with multiple mental health outcomes–is consistent with previous research that found little evidence for specificity in the effects of adverse events in the etiology of mental disorders [2,6,10,11,16]. Our results replicate and strengthen a number of prior studies revealing the broad-based impact of ACEs on depressive symptoms and drug use in early adulthood [15,37], although the association of ACEs with antisocial behavior in this period of life is to our knowledge a novel finding. While novel, this latter finding was not wholly unanticipated, as it supports Widom's assertion that childhood victimization may be an important cause of juvenile delinquency [38]. Taken together, these findings, coupled with other evidence that the impact of major childhood adversities persists well into adulthood, indicate the critical need for prevention and intervention strategies targeting early adverse experiences and their mental health consequences.

Because the transition to adulthood is a watershed developmental period, the mental health consequences of ACEs are likely to have far-reaching impact by disrupting the establishment of positive roles and relationships that set the course for adult occupational and social attainment [31,39]. However, understanding the ways in which the mental health consequences of early adversity impact both the selection of and ability to function in young adult roles may provide promising avenues for effective intervention. Because of the fluidity and malleability of roles during this period (see [40]), the transition to adulthood offers a potential "turning point" in the lives of disadvantaged youth. For example, previous research has shown that both post-secondary education and supportive romantic relationships positively influence the lifecourse trajectories of at-risk young adults [41-45]. Moreover, these roles are likely synergistic in their influence: One of the benefits of higher education in women is that it delays establishment of committed romantic relationships, resulting in higher quality marriages [43,44] which promote better mental health [46]. Clearly, strategies for preventing serious childhood adversity would be most beneficial: however, the malleability of young adulthood may provide additional opportunities to re-direct lifecourse trajectories in a positive direction and to prevent the adult mental health consequences of ACEs.

Although some gender differences in the impact of ACEs on mental health in young adults were found, our findings, taken as a whole, suggest that the contention that child abuse results in gender-typical psychopathology [47] is not so clear-cut. Young men are equally as likely as young women to exhibit depressive symptoms in response to ACEs. In addition, although the impact of ACEs on antisocial behavior was generally much stronger among young men, young women exposed to some ACEs do exhibit elevated levels of antisocial behavior. We did find one noteworthy gender difference, however. Sexual abuse/assault is associated with much higher levels of drug use, depressive symptoms and antisocial behavior in young men than in young women. Our results were based on a very small sample of sexually victimized boys, so this finding needs to be viewed with caution. Nevertheless, because the impact of sexual abuse among boys is understudied, this result underlines the need for further longitudinal research on the impact of sexual abuse/assault among boys.

To our knowledge, our study is the first to investigate racial/ethnic differences in the impact of a variety of ACEs on a variety of outcomes for the three most prevalent racial/ethnic groups in the US. Our results indicate that when racial/ethnic differences exist, young Whites consistently exhibit greater vulnerability to ACEs, particularly for externalizing behaviors. One explanation is that these results may illustrate a "steeling effect" [48] in which youths in some ethnic groups are better able to successfully cope with stress and adversity and are consequently less prone to mental health difficulties. Research on coping processes may provide support for this explanation, as there is evidence that cognitive coping styles more typical among ethnic subcultures may explain differential racial/ethnic vulnerability to stress [26]. For example, differences in coping styles partially explain greater vulnerability to PTSD among Hispanic compared to Black and White police officers (see [27]), and greater religiosity, found among Blacks compared to Whites, has been found to be protective [49].

Finally, the limitations of our study need to be noted. While our response rates were high for the original survey of respondents, as well as for the follow-up, the differential attrition of Hispanic and Black respondents relative to Whites is a limitation. In addition, despite the use of multiple waves of data to separate predictors from outcomes, the direction of causality between ACEs and mental health may be tenuous. This is particularly true for antisocial behavior and drug use, both of which may increase exposure to some types of ACEs; moreover a complicated, non-recursive relationship between ACEs and childhood antisocial/drug activity may exist (see [19] for discussion of this issue). Finally, consistent with other studies investigating the relationship between ACEs and mental health [2,6,8], we did not take into account the age at which the adversities occurred in our analysis. Although this is clearly an important factor in determining their developmental impact (see [19]), we were unable to consider age at occurrence because of the low frequency of occurrence of these ACEs in our data. This is an important avenue for future study.

## Conclusion

Our sample of young adults from urban, socio-economically disadvantaged communities reported high prevalences of adverse childhood experiences. The public health impact of childhood adversity is evident in the very strong association between childhood adversity and depressive symptoms, antisocial behavior, and drug use during the early transition to adulthood. These findings, coupled with evidence that the impact of major childhood adversities persists well into adulthood, indicate the critical need for prevention and intervention strategies targeting early adverse experiences and their mental health consequences.

## Competing interests

The author(s) declare that they have no competing interests.

## Authors' contributions

EAS: statistical analyses and manuscript writing

RHA: study design and manuscript writing

SG: study design and manuscript writing

All authors read and approved the final manuscript.

## Pre-publication history

The pre-publication history for this paper can be accessed here:


